# Exploration of Microbially Derived Natural Compounds against Monkeypox Virus as Viral Core Cysteine Proteinase Inhibitors

**DOI:** 10.3390/v15010251

**Published:** 2023-01-16

**Authors:** Amit Dubey, Maha M. Alawi, Thamir A. Alandijany, Isra M. Alsaady, Sarah A. Altwaim, Amaresh Kumar Sahoo, Vivek Dhar Dwivedi, Esam Ibraheem Azhar

**Affiliations:** 1 Computational Chemistry & Drug Discovery Division, Quanta Calculus, Greater Noida 201310, India; 2 Special Infectious Agents Unit-BSL3, King Fahd Medical Research Center, King Abdulaziz University, Jeddah 21362, Saudi Arabia; 3 Department of Medical Microbiology and Parasitology, Faculty of Medicine, King Abdulaziz University Hospital, Jeddah 21362, Saudi Arabia; 4 Infection Control and Environmental Health Unit, King Abdulaziz University Hospital, Jeddah 21362, Saudi Arabia; 5 Department of Medical Laboratory Sciences, Faculty of Applied Medical Sciences, King Abdulaziz University, Jeddah 21362, Saudi Arabia; 6 Department of Applied Sciences, Indian Institute of Information Technology Allahabad, Allahabad 211015, India; 7 Bioinformatics Research Division, Quanta Calculus, Greater Noida 201310, India

**Keywords:** monkeypox virus, natural products, antivirals, molecular dynamics, virtual screening

## Abstract

Monkeypox virus (MPXV) is a member of the *Orthopoxvirus* genus and the *Poxviridae* family, which instigated a rising epidemic called monkeypox disease. Proteinases are majorly engaged in viral propagation by catalyzing the cleavage of precursor polyproteins. Therefore, proteinase is essential for monkeypox and a critical drug target. In this study, high-throughput virtual screening (HTVS) and molecular dynamics simulation were applied to detect the potential natural compounds against the proteinase of the monkeypox virus. Here, 32,552 natural products were screened, and the top five compounds were selected after implementing the HTVS and molecular docking protocols in series. Gallicynoic Acid F showed the minimum binding score of −10.56 kcal/mole in the extra precision scoring method, which reflected the highest binding with the protein. The top five compounds showed binding scores ≤−8.98 kcal/mole. These compound complexes were tested under 100 ns molecular dynamics simulation, and Vaccinol M showed the most stable and consistent RMSD trend in the range of 2 Å to 3 Å. Later, MM/GBSA binding free energy and principal component analysis were performed on the top five compounds to validate the stability of selected compound complexes. Moreover, the ligands Gallicynoic Acid F and H2-Erythro-Neopterin showed the lowest binding free energies of −61.42 kcal/mol and −61.09 kcal/mol, respectively. Compared to the native ligand TTP-6171 (ΔG_Bind_ = −53.86 kcal/mol), these two compounds showed preferable binding free energy, suggesting inhibitory application against MPXV proteinase. This study proposed natural molecules as a therapeutic solution to control monkeypox disease.

## 1. Introduction 

Monkeypox is an emerging infectious disease caused by the monkeypox virus (MPXV), which is transmitted to humans from infected rodents. Prior to the monkeypox disease’s outbreak in April 2022, human cases of monkeypox infection were primarily reported in African countries. MPXV caused multiple outbreaks in Central and West Africa [1,2], and occasionally in Europe and North America [2]; 47 human cases were reported in the US Midwest in 2003 [3]. In recent reports published by the WHO (World Health Organization), there were a number of cases reported among the non-endemic regions, including Europe (88%), America (10%), the Eastern Regions (1%), and the Western Pacific Regions (<1%) [4]. According to recent WHO reports, the number of cases globally increased by 15% between August 20 and August 26, 2022 [4]. Detailed information about this disease is still limited, including its transmission, risk factors, clinical representation, and outcomes. Human monkeypox virus was first reported in Africa in 1970 in the Democratic Republic of Congo (known as Zaire) [5,6]. The literature review stated that the MPXV strain differs from previous strains after 1997 [7]. MPXV is a zoonotic double-stranded DNA virus related to the variola major virus (VACV), cowpox virus (CPXV), and vaccina virus (VACV) [8,9,10,11]. The monkeypox virus belongs to the genus *Orthopoxvirus* within the *Poxviridae* family. 

The MPXV infection is spread through large respiratory droplets, salivary secretions, close or direct contact with skin lesions, and contaminated objects or fomites by infected individuals [12,13,14]. According to studies conducted in the 1970s, routine smallpox immunization can reduce the risk of developing the disease by up to 90% after exposure to MPXV [15,16]. On 9 August 2022, the FDA authorized JYNNEOS as the sole emergency vaccine for the treatment of monkeypox [17]. Contrary to vaccinations, which have a delayed protective effect, the use of antiviral medications for the treatment of already infected individuals would have an immediate impact. There are currently no known FDA-approved medications for treating monkeypox. However, to treat monkeypox patients with severe symptoms during the emergency situation of the 2022 outbreak, the FDA approved Tecovirimat as the only antiviral drug to inhibit the growth of MPXV [12,18]. Noticeably, Cidofovir, Brincidofovir, and Tecovirimat were approved for the treatment of smallpox, though they also exhibited activity against MPXV. Tecovirimat is much more specific for *orthopoxviruses* and prevents the formation of the enveloped virus, whereas Cidofovir and Brincidofovir inhibit DNA replication and exhibit activity against different dsDNA viruses [19].

The production of infectious progeny is dependent on the proteolytic maturation of the core proteins of the *orthopoxvirus* [20]. Poxviruses often replicate in the cytoplasm of infected cells, unlike other DNA viruses, by employing a variety of virus-encoded proteins such as cysteine proteinase [21]. Proteases, which cleave precursor polyproteins, are central to the viral replication cycle. Inhibiting viral protease with active chemical substances is a feasible therapeutic strategy as these enzymes are considered as essential for viral replication [22]. The primary structural and membrane proteins of viruses are cleaved by the cysteine proteinase known as (I7L) core proteinase [23]. As per their essential role in viral replication by cleaving precursor polyproteins [22], proteases are known as most promising therapeutic targets, and protease inhibitors in other viruses, including HIV, have shown promising viral protease inhibition [24]. TTP-6171 has been identified as an I7L inhibitor [25]. However, mutations could also produce drug-resistant I7L [23]. Fosdagrocorat and Lixivaptan were investigated in a recent study targeting cysteine proteinase of MPXV [25]. Another study examined the computational repurposing drug design studies for tetracycline to target the cysteine proteinase [26]. 

In this study, a comprehensive computational pipeline has been applied to identify potential hit compounds against MPXV proteinase. The structure of the protein was modeled, and using the molecular dynamics simulation, it was further refined to generate the most probable conformation of the protein. NP_ATLAS database was screened against the modeled protein structure in multiple phases to screen natural products. Eventually, the top five drug-like candidates were selected for the explicit 100 ns molecular dynamics simulation. Dynamic characteristics of the protein–ligand complexes were studied using RMSD, RMSF, MMGBSA, and PCA to conclude the binding affinity and stability of the hit compounds against MPXV proteinase.

## 2. Methodology

### 2.1. Protein Structure Modeling

The 3D structure of the MPXV proteinase has not been experimentally solved. Therefore, the structure of proteins was computationally modeled in this study. The gene accession ID NP_536495.1 was used to get the MPXV proteinase primary sequence in FASTA format from the GenPept database [27]. Later, the structure of protein was modeled using Alphafold Colab v2.1.0, which is a novel machine learning approach to predict 3D structures that uses multiple sequence alignment in the deep learning algorithm to integrate biological and physical information about protein structure [28]. This structure was further validated using molecular dynamics (MD) simulation.

### 2.2. Modeled Structure Dynamics Simulation

The modeled protein structure was simulated for 100 ns using the free academic program Desmond [29,30]. Protein was prepared using the Schrodinger suite protein preparation wizard module with evasion settings, followed by the generation of an orthorhombic box as a simulation box with the system building tool. Salt and ion placement were omitted at 20 Å around ligand binding sites, and the entire system was immersed in a water bath with sodium as counter ions and a Monte Carlo equilibrated periodic transferable intermolecular potential with 4 points (TIP4P) water model. The constant pressure was ensured for MD simulation by assigning 0.002 ps time steps for anisotropic diagonal position scaling. Furthermore, the temperature was slowly increased to 310 K, along with a 20 psi NPT reassembly at 1 atm pressure. Compactness of the whole simulation system was preserved at 1 g/cm^3^, and MD calculations were performed in the academic version of the Desmond program at the default force field, which is optimized potential for liquid simulations in (OPLS)-2005 [29,30]. The frames resulting from each trajectory were clustered based on RMSD to get representative conformations of the system. The Desmond Trajectory Clustering tool implemented in Maestro suite performs the clustering process. The most populated middle structure was selected from the cluster. This structure was further used for binding pocket prediction and virtual screening. 

### 2.3. Binding Grid Box

Here, the Computed Atlas of Surface Topography of proteins (CASTp) server predicted the MPXV proteinase-binding pocket. The CASTp web server predicts the binding cavity of a protein structure with the help of geometrical and topological properties [31]. The best pocket detected by the CASTp was considered for generating the grid box; this pocket had an area of 877.44 Å^2^ and a volume of 930.93 Å^3^. The target protein’s 3D-modeled structure, the MPXV proteinase, was docked with the natural products extracted from NP_ATLAS. The grid box was designed using the binding pocket residues that were detected by the CASTp server. The binding sites were covered by a grid box with dimensions 60 Å × 60 Å × 60 Å in the x, y, and z axis, with the center of the box at 23.95 Å, −7.71 Å, and −11.58 Å.

### 2.4. Compound Library 

The compound library was generated using the NP_ATLAS database, which is a database of microbial natural products that facilitates the development of new therapeutic molecules. The database contain 24,594 compounds with their structure, names, source organism, and isolation references, and total syntheses are an instance for structural reassignment [32]. Moreover, recently in NP_ATLAS 2.0 new compounds were added that made its compound count 32,552;full taxonomic data were also added along with chemical ontology terms [33].

### 2.5. Ligand Preparation

The compounds (ligands) were prepared using Schrodinger LigPrep [34,35] where the tool generated precise, energy-optimized 3D molecular structures. It also applied advanced algorithms to reduce Lewis structures and eliminate shortcomings in the ligands to minimize computational errors. It produced broad chemical structural diversity through the input of a single structure by expanding the tautomeric state, the ionization state, and ring conformations. LigPrep applied a filter to eliminate compounds that do not meet users’ specific criteria. In addition, LigPrep processed nearly one ligand every second, enabling the conversion of a whole database. Here in this study, the QikProp filter was used to filter out the compounds that did not follow the ADME and Lipinski filter criteria, and compounds were obtained [36]. Later, high-throughput virtual screening (HTVS) was applied using the predicted 3D MPXV proteinase against the compounds retrieved from the QikProp filter.

### 2.6. Virtual Screening

**Figure 1:**
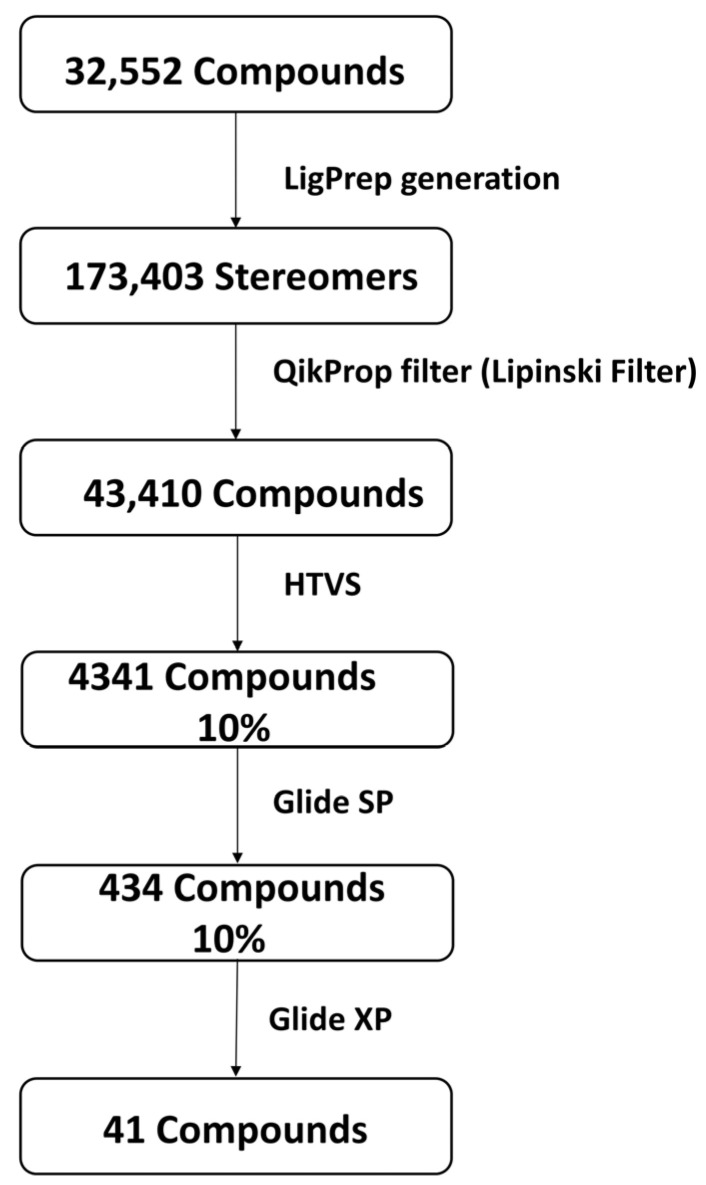
Phases of compound screening along with compound (ligands) preparation using Schrödinger Release 2021–3 packages.

Figure 1 shows the preparation of the compounds and the virtual screening workflow. In this study, structure-based virtual screening was performed using Schrödinger Glide high-throughput virtual screening (HTVS) with Glide standard precision (SP) and Glide extra precision (XP) [37,38,39,40]. The SP mode reliably docks tens to thousands of ligands with high accuracy, and the XP (extra precision) mode provides further elimination of false positives through more extensive sampling and an advanced scoring function. Glides used a series of hierarchical filters to search for potential ligand sites in the active site area of receptors. In addition, on a grid, the shape and features of the receptor were signified by various sets of fields that offered increasingly precise scoring of the ligand pose [37]. 

### 2.7. Protein–Ligand MD Simulation 

The top five compound complexes were selected from the virtual screening, and molecular docking underwent 100 ns of MD simulation for the best docked poses using the Desmond module of the Schrodinger suite [41]. All conditions used in the MD simulation of the modeled protein structure were similarly applied to the protein–ligand docked complexes. The procedure has been explained in Section 2.2 of the methodology. The simulation interaction diagram tool in the free academic Desmond–Maestro interpolarity tool was used to investigate the simulation trajectories. The MD trajectory obtained for each complex was evaluated by converting it to a Bio3D-compatible format to estimate the principal component analysis (PCA) using the “R” program [42,43]. The generated trajectories with coordinates every 20 ps for the chosen complexes were evaluated using the Simulation Interaction Diagram of Desmond in the Schrödinger package. Here, the RMSD, RMSF, and protein–ligand interaction profile were extracted from the 100 ns MD simulation trajectory of each complex. The Bio3D tool was used for additional post-dynamics calculations.

### 2.8. MM/GBSA Calculation

The binding free energy module of the MM/GBSA protocol in the Schrodinger suite [44] was used to calculate the end-point binding free energy for each simulated protein–ligand complex. Here, MM/GBSA calculations were performed using the Schrodinger suite 2019–2 Prime module, OPLS-2005 force field [45] with default parameters on the derived frame (structure) from the 100 ns MD simulation trajectory of each protein–ligand system. MM/GBSA calculated the energy components for proteins, ligands, and protein–ligand complexes. This includes the free receptor/protein, free ligand, the complex, receptor from the complex, and ligand from the complex. Overall, the MM/GBSA binding free energy is represented by the equation shown in (1).

(1) $$\Delta G_{bind}^{MMGBSA}=E_{complex}-\left(E_{receptor}^{complex}+E_{ligand}^{complex}\right)\;$$

## 3. Results and Discussions

### 3.1. Binding Pocket and Active Site of Proteinase

Previous studies showed five residues in the “core” binding site motif (W-HW-Q-C) of the I7L [46]. The catalytic motif Cys^328^ and His^241^ is present in the core active site of I7L. In addition to the backbone amino group of Cys^328^, the Gln^322^ side chain produces a subtilisin-like oxyanion hole. Side chains Trp^242^ and Trp^168^ also contribute to the formation of a narrow channel through which the substrate can enter. Here, the CASTp result of the protein of MPXV showed that the active site residue Cys^328^ was present in the binding pocket. The residues listed in the best binding pockets detected by CASTp were Met^1^, Glu^2^, Arg^3^, Tyr^4^, Asp^6^, Leu^7^, Ser^10^, Phe^17^, Thr^18^, Leu^21^, Ser^128^, Lys^129^, Ile^131^, Gln^135^, Met^136^, Met^159^, Lys^160^, Ile^161^, Pro^163^, Glu^164^, Phe^236^, Cys^237^, Tyr^238^, Leu^239^, Ser^240^, Trp^242^, Lys^243^, Asp^258^, Gly^260^, Gly^261^, Asn^262^, Ile^263^, Glu^266^, Phe^278^, Ser^279^, Asp^292^, Thr^294^, Asn^295^, Asp^297^, Ile^298^, Val^318^, Glu^319^, Val^320^, Asn^321^, Gln^322^, Leu^323^, Leu^324^, Glu^325^, Ser^326^, Cys^328^, Phe^331^, Phe^357^, Lys^358^, Phe^359^, Leu^360^, Ala^361^, and Asp^362^. The active site residues were mapped with the best binding site detected by the CASTp server, which authenticated the application of the binding site for the virtual screening and molecular docking.

### 3.2. Virtual Screening Analysis

The most populated cluster obtained after the 100 ns MD simulation was used to extract the middle structure, which was used further for virtual screening against the natural compound dataset sourced from the NP-ATLAS database. The virtual screening employs computer-based techniques to identify novel ligands based on their 3D structure’s complementarity and binding [47]. Glide suite was used for virtual screening with the QikProp filter, where the ADME and Lipinski criteria were evaluated for the small compounds. Here, 32,552 compounds retrieved from NP-ATLAS database [33] were originally screened against the MPXV cysteine proteinase. Later, LigPrep was used that generated 173,403 stereoisomers; then, these stereoisomers were filtered using the QikProp filter (Lipinski filter). This cut down the number to 43,410 compounds. Initially, these compounds were screened using a high-throughput virtual screening (HTVS) protocol, and 10% of the top compounds (4341) based on the binding score were selected for the next phase of docking. In the second phase of screening, the top 10% of compounds from HTVS were further docked using Glide and scored based on standard precision (SP), which further assisted in selecting 434 (10%) compounds. Later, 10% of the top-ranked compounds in SP scoring function were eventually docked and scored with the extra precision (XP) scores. Table 1 shows the final set of 41 compounds selected by Glide and scored on the XP scoring function. These structures were ranked based on their binding scores, and the top five compounds were selected for further evaluation. The reference ligand TTP-6171 was also docked with the proteinase MPXV. The 2D structure of the reference ligand is shown in the Supplementary Figure S1. 

**Figure 2:**
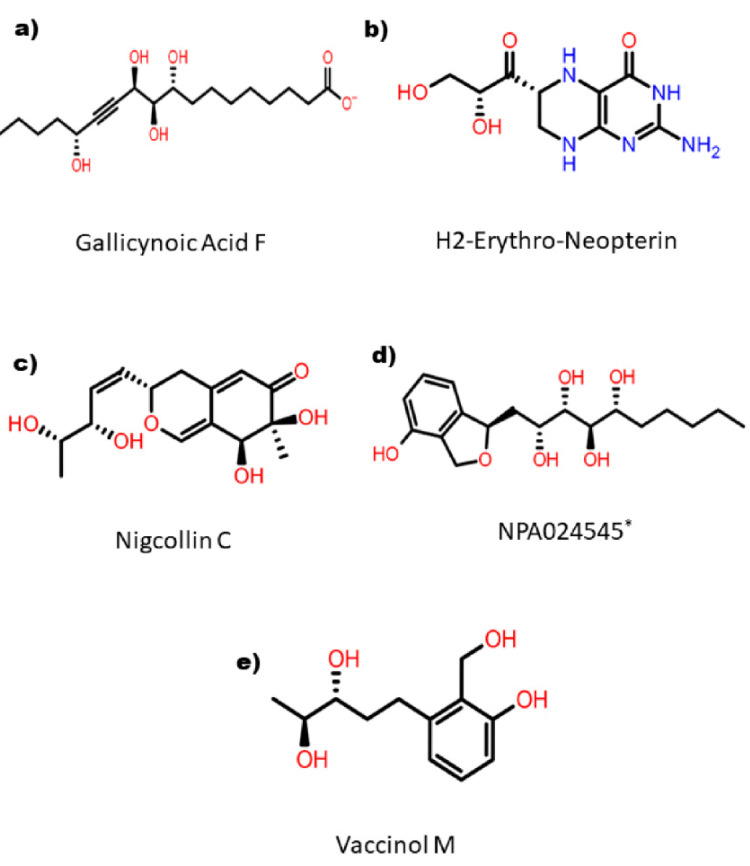
2D representation of the selected top five hit compounds derived after series of screening methods implemented on natural compounds against MPXV proteinase. * Name of the compound is not available.

The top five compounds were selected that showed binding XP scores of ≤ −8.98 kcal/mol. These top hits were represented in their corresponding 2D structure format, as shown in Figure 2. These compounds were (1) Gallicynoic Acid F (NPA002071), (2) H2-Erythro-Neopterin (NPA000530), (3) Nigcollin C (NPA029767), (4) NPA24545, and (5) Vaccinol M (NPA030378). Gallicynoic Acid F showed the lowest binding score of −10.56 kcal/mol in the XP scoring method. The compounds H2-Erythro-Neopterin, Nigcollin C, and NPA24545 showed similar binding energies of −9.64, −9.25, and −9.18 kcal/mol, respectively, while Vaccinol M showed a binding score of −8.98 kcal/mole. Interestingly, the chemical scaffolds of these top hits were not similar, either in chemical nature or in topology. Gallicynoic Acid F is an aliphatic compound with multiple hydroxyl groups and a terminal carboxyl group. However, the other four hit compounds consisted of cyclic groups. Vaccinol had the simplest chemical scaffold compared to the other three compounds. A hydrophobic straight open chain was present in Gallicynoic Acid F and NPA24545, while Vaccinol M also had a small open hydrophobic chain.

### 3.3. Intermolecular Interaction Analysis

**Figure 3:**
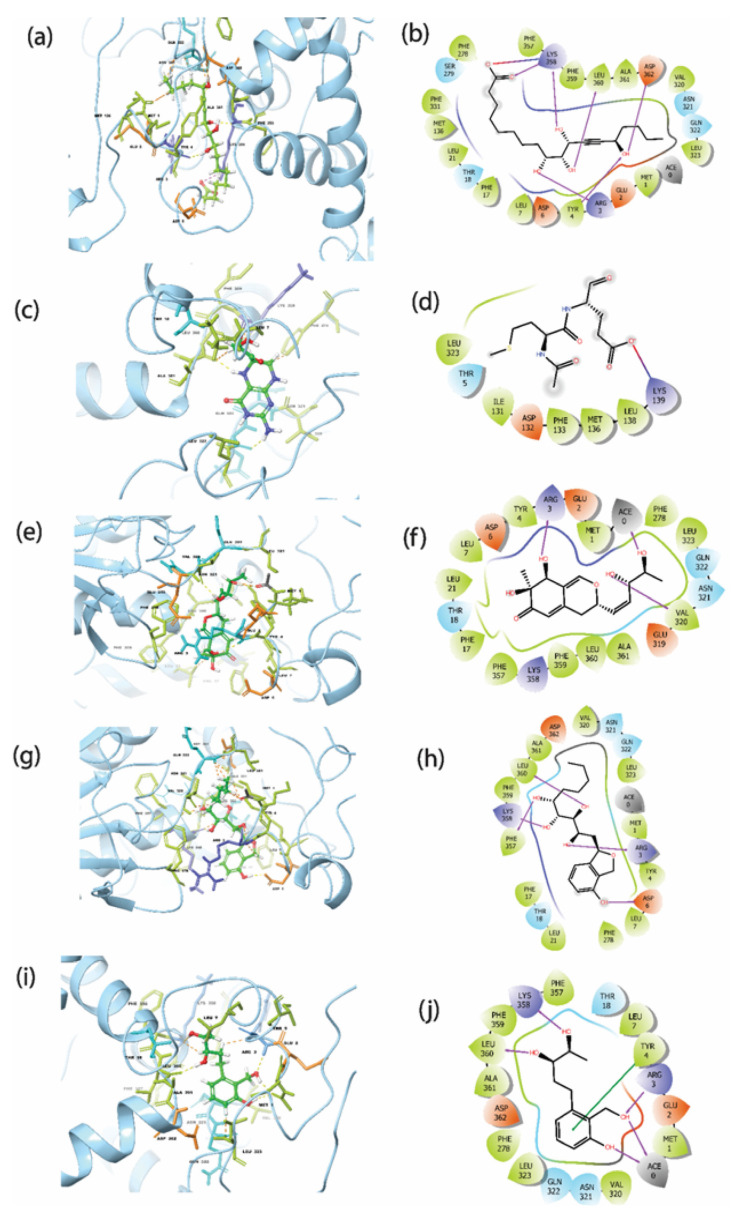
3D and 2D interaction profile of the MPXV cysteine protease with the top screened natural compounds (**a**,**b**) Gallicynoic Acid F, (**c**,**d**) H2 Erythro-Neopterin, (**e**,**f**) Nigcollin C, (**g**,**h**) NPA024545, and (**i**,**j**) Vaccinol M.

The top five hits were further studied for the protein–ligand intermolecular interaction analysis. Figure 3b showed the residues Arg^3^, Tyr^4^, Lys^358^, Leu^360^, and Asp^362^ of the protein had direct hydrogen bond formation with Gallicynoic Acid F. Here, Lys^358^ residue showed three hydrogen bonds with Gallicynoic Acid F. The ligand H2 Erythro-Neopterin had one residue, Lys^139^, which showed a hydrogen bond with the protein, as shown in Figure 3d. Another ligand, Nigcollin C, had a hydrogen bond formed with the residues Arg^3^ and Val^320^, as shown in Figure 3f. Similarly, residues Leu^360^, Lys^358^, Arg^3^, Asp^6^, and Phe^357^ had hydrogen bond interactions with the ligand NPA024545, as depicted in Figure 3h. Figure 3j shows that residues Lys^358^, Leu^360^, and Arg^3^ showed hydrogen bond interactions, and Tyr^4^ showed π-π stacking with ligand Vaccinol M. Overall, it was observed that Gallicynoic Acid F and NPA024545 showed the maximum number of hydrogen bonds. Moreover, the reference ligand TTP-6171 had no hydrogen bond detected, but it formed stacking interactions with residues and the protonated form of histidine His^23^ (as shown in Supplementary Figure S2b). This demonstrated that there was no formation of directed hydrogen bonds between the reference ligand and the protein, which may result in considerably weaker binding compared to the top hits selected. Table 2 summarizes the interaction of the protein residues with the selected top five hits along with the reference ligand TTP-6171. These interactions include the hydrogen bond, hydrophobic contacts, polar interaction, π-π stacking, and positive/negative interactions between the protein and ligands. Hydrophobic contacts were observed with Tyr^4^, Leu^7^, Phe^278^, Phe^357^, Val^320^, Leu^323^, Phe^359^, and Leu^360^ residues for all the ligands. However, except for H2-Erythro-Neopterin, Met^1^ residue was found to be involved in hydrophobic contacts for all the hit compounds. Moreover, the native ligand showed hydrophobic contacts with Leu^27^, Ile^34^, Val^36^, Leu^40^, Phe^356^, Phe^368^, Ile^371^, and Tyr^393^, which was not observed in the studied ligand complexes. Moreover, Lys^358^ showed π-π stacking with Gallicynoic Acid F, and Tyr4 showed stacking with H2-Erythro-Neopterin ligand complexes. These top five hits were analyzed for the binding free energies over the simulation trajectory to compute the more precise affinity between protein and ligands. Figure 3b,c,e,g,i shows the 3D representation of the protein–ligand interaction. Here, it was observed that the top five compounds were bound in the same binding pocket at a similar location to the protein. The Supplementary Figure S2a shows the 3D representation of the native ligand TTP-6171. Comparative study showed that the top five ligands were bound in the same binding pocket location where the native was bound.

### 3.4. Molecular Dynamic Simulation Analysis

**Figure 4:**
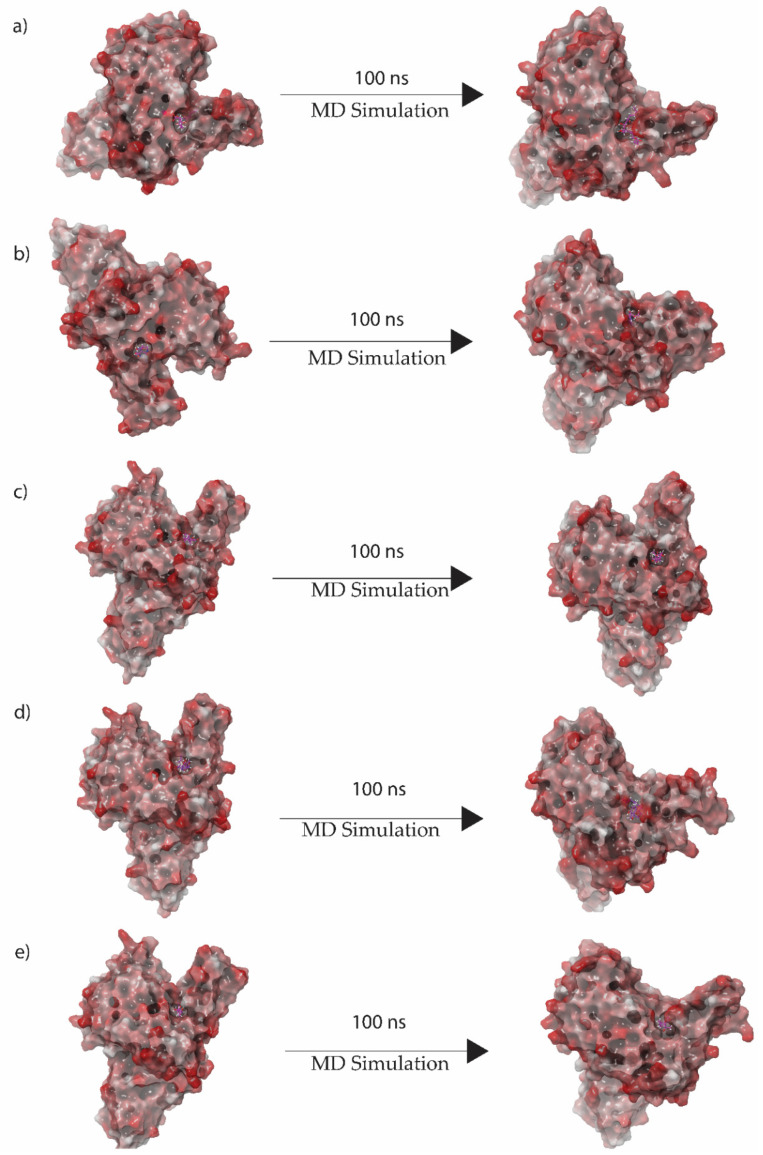
3D position of initial and final docked poses of (**a**) Gallicynoic Acid F, (**b**) H2 Erythro-Neopterin, (**c**) Nigcollin C, (**d**) NPA024545, and (**e**) Vaccinol M extracted from the 100 ns MD simulation.

On the basis of a general model of the physics of the principal interatomic interactions, molecular dynamics (MD) simulations were used to predict the movement of each atom in a system over the given period of time [48]. Therefore, selected protein complexes were monitored for docked complex stability under 100 ns MD simulations conducted in the Desmond suite. In protein–ligand complexes, the root mean square deviation (RMSD) for the MD trajectory provides critical details regarding the system flexibility. Figure 4 shows 3D structure formation of first and last docked pose. Here it was observed that the final pose varies from the initial pose both in rotational and translational space. Similarly, the final pose of the native ligand complex’s 3D structure showed positional change compared to the initial pose, as shown in Supplementary Figure S7.

#### 3.4.1. Root Mean Square Deviation (RMSD)

**Figure 5:**
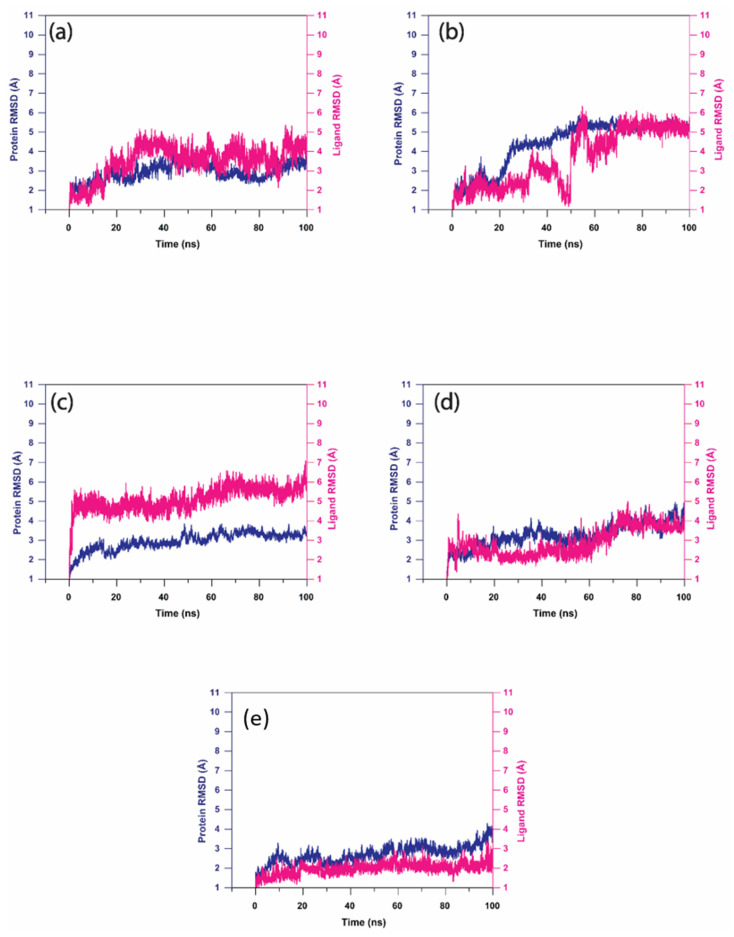
RMSD analysis on the docked viral proteins and ligands, i.e., natural compound trajectories (**a**) Gallicynoic Acid F, (**b**) H2 Erythro-Neopterin, (**c**) Nigcollin C, (**d**) NPA024545, and (**e**) Vaccinol M.

In this analysis, RMSD was calculated to quantify the conformational changes of the protein and ligand shown after forming the complex. Figure 5 shows the RMSD of the proteins and ligands in the top five hit compound complexes. The RMSD of Gallicynoic Acid F in the protein–ligand complex showed deviation at 18 ns where the RMSD ranged from 3.5 Å and reached 5 Å after 30 ns of the simulation. Later, the RMSD dropped to 3.5 Å after 45 ns of the simulation, while it showed fluctuation between 3.5 Å to 4.5 Å for the rest of the simulation. The protein in the complex of Gallicynoic Acid F showed a similar trend, with lower RMSD range of 2.5 Å to 3.5 Å, as shown in Figure 5a. In the H2 Erythro-Neopterin complex, both the protein and ligand showed stable RMSD of 2 Å for the first 20 ns of the simulation, where the protein took a steep rise to 5 Å at 25 ns and stayed consistent for the rest of the 100 ns MD simulation. Similarly, the ligand of H2 Erythro-Neopterin showed fluctuation in RMSD from 30 ns to 70 ns, while it finally showed stability at 5 Å for the last 30 ns of the MD simulation, as shown in Figure 5b. Nigcollin C-protein complex showed that the protein had stable and consistent RMSD in the range of 2.5 Å to 3.5 Å, while the ligand showed consistent RMSD in the range of 5 Å to 6.5 Å during the 100 ns MD simulation, while it reached 7 Å during the 100 ns simulation, as shown in the Figure 4c. The protein in the complex with NPA024545 showed stable RMSD in the range of 2 Å to 3 Å until 70 ns during the MD simulation, while it reached 4 Å with stable and consistent RMSD for the rest of the simulation. However, the ligand NPA024545 showed fluctuation for the first 20 ns of the simulation and stayed stable and consistent from 20 ns to 60 ns of the simulation, with 2 Å RMSD; later, it showed a steep rise to 4 Å, where it stayed consistent for the rest of the 100 ns simulation (Figure 5d). The Vaccinol M protein and ligand complex showed a similar trend, with a stable and consistent RMSD. Here, the protein showed RMSD in the range of 2.5 Å to 3 Å for a 75 ns duration of the MD simulation, while it reached 4 Å at the end of the 100 ns simulation. The ligand Vaccinol M showed the most stable and consistent RMSD in the range of 2 Å to 3 Å for the 100 ns MD simulation, as shown in the Figure 5e. Moreover, in Supplementary Figure S3a, TTP-6171 RMSD showed the protein is stable and consistent at 4 Å compared to the initial conformation, and the ligand showed deviation continuously in the range of 2 Å to 5.5 Å for the first 30 ns of the MD simulation. Later, it increased and fluctuated in the range of 8 Å to 10 Å RMSD from 30 ns to 85 ns during the MD simulation and showed stability at 7 Å. 

#### 3.4.2. Root Mean Square Fluctuation (RMSF)

RMSF values were determined for the protein and ligand molecules to account for specific variations of each residue/atom. Supplementary Figure S4 showed the RMSF of protein from the protein–ligand complex in all the top five hit compound complexes. The RMSF of the protein showed similar lower fluctuations throughout the MD simulation in all complexes. However, at the 150 residue and 400 residue positions, it showed two peaks with RMSF > 3 Å for all the top five ligands. The RMSF of the docked ligand is shown in Supplementary Figure S5. Here, the ligands Nigcollin C and Vaccinol M showed fluctuations with <2 Å RMSF with three peaks, as shown in Figure S5c,e. The ligand NPA024545 showed RMSF in the range > 3 Å for the terminal atoms, while it showed two peaks, as shown in Figure S5d. The ligands Gallicynoic Acid F and H2 Erythro-Neopterin showed one peak each with RMSF > 3 Å. Supplementary Figure S3b,c show the RMSF of the protein and native ligand, respectively. Here, the native ligand showed two peaks, with RMSF > 3 Å for the protein, while for the ligand all the atoms showed RMSF > 3 Å. 

### 3.5. MD Simulation Protein Ligand Interactions Mapping

**Figure 6:**
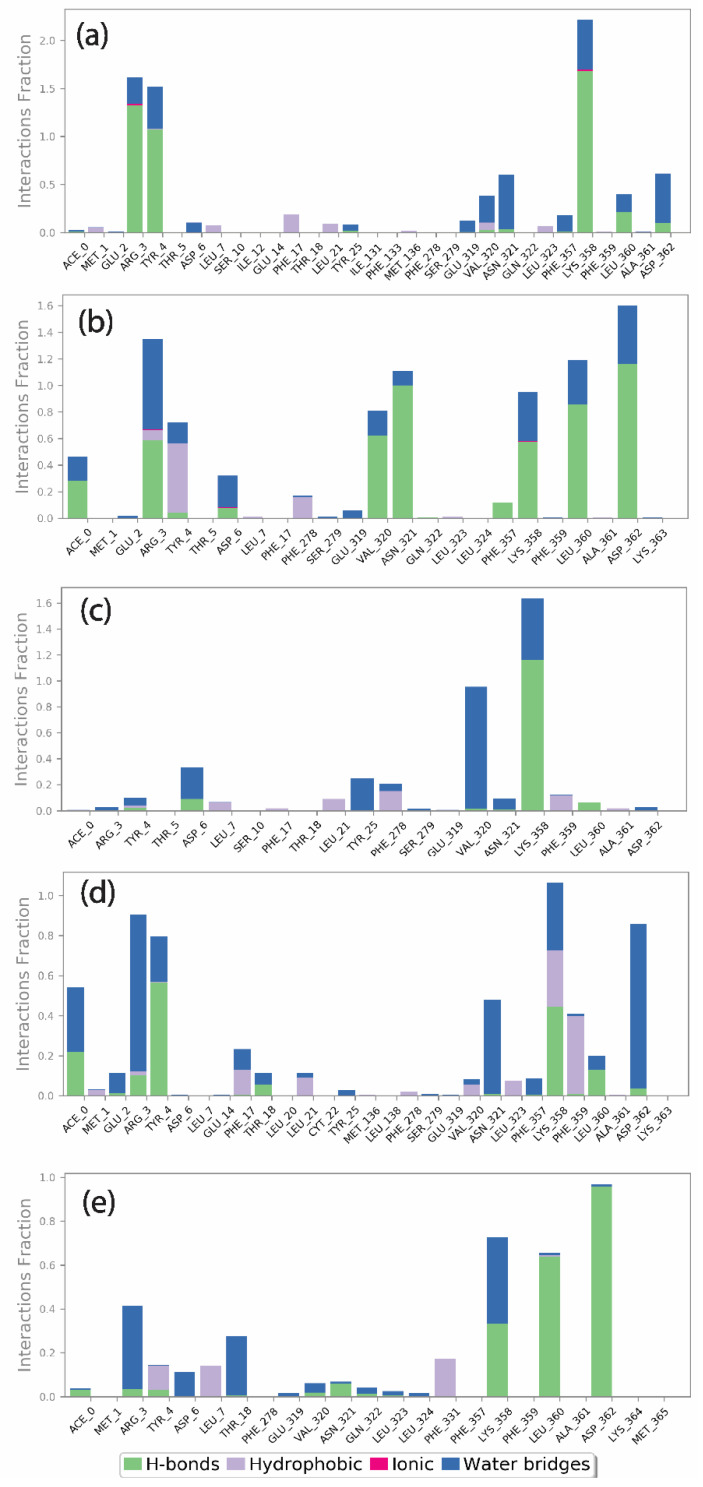
Protein–ligand interactions mapping for viral protein with selected natural compounds over the 100 ns simulation. (**a**) Gallicynoic Acid F, (**b**) H2 Erythro-Neopterin, (**c**) Nigcollin C, (**d**) NPA024545, and (**e**) Vaccinol M.

Furthermore, the mapping of protein–ligand interactions for the viral proteins with top screened natural compounds is shown in Figure 6. The protein–ligand interaction map for the Gallicynoic Acid F complex showed the presence of Met^1^, Glu^2^, Arg^3^ Tyr^4^, Thr^4^, Asp^6^, Leu^7^, Ser^10^, Ile^12^, Glu^14^, Phe^17^, Thr^18^, Leu^21^, Tyr^25^, Ile^131^, Phe^133^, Met^136^, Phe^278^, Ser^279^, Glu^319^, Val^320^, Asn^321^, Gln^322^, Leu^323^, Phe^357^, Lys^358^, Phe^359^, Leu^360^, Ala^361^, and Asp^362^ neighboring interacting residues. As shown in Figure 6a, the molecules formed hydrogen bonds, hydrophobic contacts, ionic interactions, and water bridges. Arg^3^, Tyr^4^, and Lys^358^ contributed to a higher ratio for hydrogen bonds followed by water bridges with a low ratio of interaction. The H2 Erythro-Neopterin compound interaction showed the contribution of Met^1^, Glu^2^, Arg^3^, Tyr^4^, Thr^5^, Asp^6^, Leu^7^, Phe^17^, Phe^278^, Ser^279^, Glu^319^, Val^320^, Asn^321^, Gln^322^, Leu^323^, Leu^324^, Phe^357^, Lys^358^, Phe^359^, Leu^360^, Ala^361^, Asp^362^, and Lys^363^ residues in the formation of hydrogen bonds, hydrophobic contacts, and water bridging. Here, Asn^321^, Leu^360^, and Asp^362^ showed a higher ratio for hydrogen bond formation and Arg^3^, Val^320^, and Lys^358^ showed a relatively lower hydrogen bond interaction fraction compared to them. The residues Arg^3^ showed an equal ratio of hydrogen bonds and water bridge interaction fraction, while the residues Asn^321^, Leu^360^, Asp^362^, Val^320^, and Lys^358^ showed a comparatively lesser water bridge interaction fraction, as shown in Figure 6b. Nigcollin C complex showed the contributions of Arg^3^, Tyr^4^, Thr ^5^, Asp^6^, Leu^7^, Ser^10^, Phe^17^, Thr^18^, Leu^21^, Tyr^25^, Phe^278^, Ser^279^, Glu^319^, Val^320^, Asn^321^, Ljus^358^, Phe^359^, Leu^360^, Ala^361^, and Asp^362^ neighboring interacting residues. Val^320^ and Lys^358^ showed maximum contributions in water bridging and hydrogen bonding, respectively, as shown in Figure 6c. Here, Val^320^ showed relatively higher water bridge interaction fraction, while Lys^358^ showed a higher hydrogen bond interaction ratio compared to the water bridge interaction fraction. Similarly, the NPA024545 complex has Met^1^, Glu^2^, Arg^3^, Tyr^4^, Asp^6^, Leu^7^, Glu^14^, Phe^17^, Thr^18^, Leu^20^, Leu^21^, Tyr^22^, Met^136^, Leu^138^, Phe^278^, Ser^279^, Glu^319^, Val^320^, Asn^321^, Leu^323^, Phe^357^, Lys^358^, Phe^359^, Leu^360^, Ala^361^, Asa^362^, and Lys^363^ neighboring interacting residues. Here, a relatively higher interaction fraction was observed for Arg^3^, Asn^321^, and Asp^362^ in water bridge bond formation, while Tyr ^4^ and Lys^358^ showed a comparatively higher hydrogen bond interaction ratio. Here, Phe^359^ and Lys^358^ also showed hydrophobic interaction ratios as shown in Figure 6d. Finally, the Vaccinol M complex has Met^1^, Arg^3^, Tyr^4^, Asp^6^, Leu^7^, Thr^18^, Phe^278^, Glu^319^, Val^320^, Asn^321^, Gln^322^, Leu^323^, Leu^324^, Phe^331^, Phe^357^, Lys^358^, Phe^359^, Leu^360^, Ala^361^, Asp^362^, Lys^364^, and Met^365^ as neighboring interacting residues. It was observed that Leu360 and Asp362 showed relatively higher hydrogen bond interaction fractions, while Lys358 showed equal ratios of hydrogen bond and water bridge interactions. Here, Arg4 and Thr18 showed a comparatively higher water bridge interaction fraction, as shown in Figure 6e. 

In addition to this, the native ligand TTP-6171 showed a higher ratio of hydrophobic interactions with His^23^ and Leu^27^ and relatively lower hydrophobic interaction fraction with Phe^356^, Phe^368^, and Ile^371^. In addition, the residues Ile^34^, Val^36^, Tyr^393^, Leu^415^, and Arg^417^ showed hydrogen bond interactions, hydrophobic interaction, and water bridge interaction, as shown in Supplementary Figure S6a. Moreover, as compared to the top five interaction fractions, hydrophobic interactions were dominated in the native ligand complex, whereas in the top screened compounds, hydrogen bond interactions and water bridges were maximally observed.

**Figure 7:**
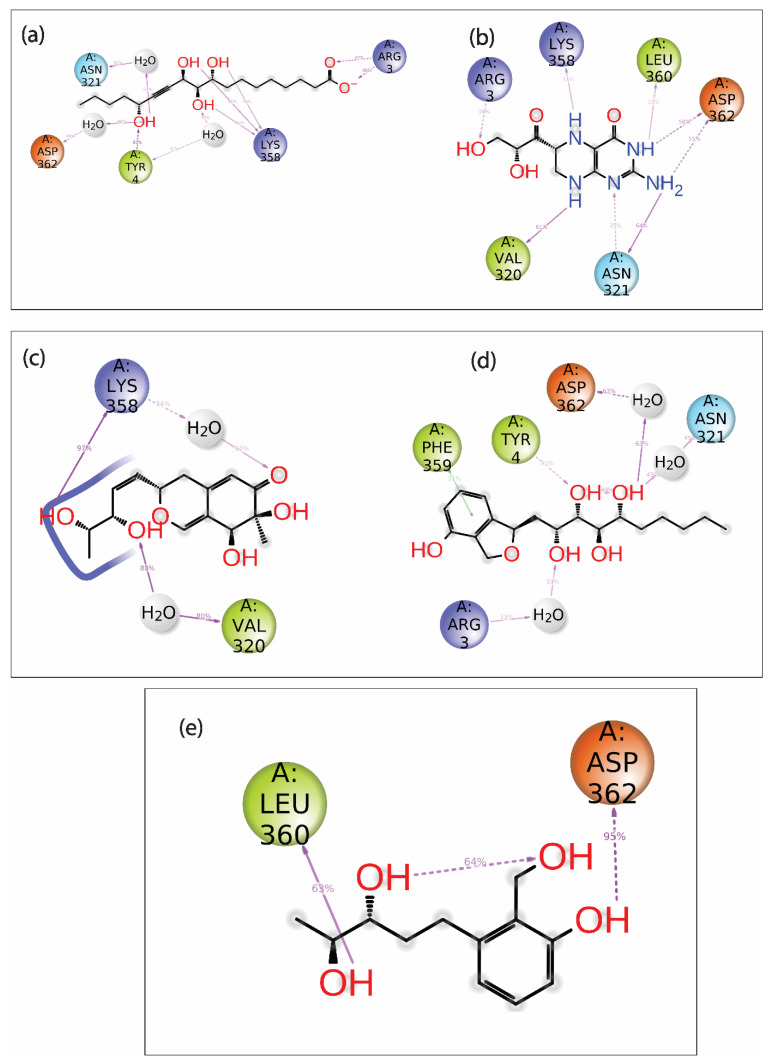
2D interaction diagram of protein–ligand contact for viral protein with selected natural compounds, i.e., (**a**) Gallicynoic Acid F, (**b**) H2 Erythro-Neopterin, (**c**) Nigcollin C, (**d**) NPA024545, and (**e**) Vaccinol M.

Additionally, the protein–ligand intermolecular interaction was calculated for the top five hits. Here, the 2D representation of the interaction of the protein and ligand was extracted from the frames generated during the 100 ns MD simulation and is shown in Figure 7. The compound Gallicynoic Acid F showed multiple hydrogen bond interactions with five residues, while the residue Lys^358^ showed three hydrogen bond interactions with 51%, 40%, and 41% of the total frames generated during the simulation. The residue Try^4^ showed a relatively higher hydrogen bond interaction with 93% of total frames, while Asp^362^ and Asn^321^ showed water-mediated hydrogen bonding with 48% and 52% of the total frames, respectively. Here, Arg^3^ also showed two hydrogen bonds in 63% and 68% of the total frames, respectively. The protein–ligand complex with the compound H2 Erythro-Neopterin during the simulation showed six hydrogen bonds with Arg^3^, Lys^358^, Leu^360^, Asp^362^, Val^320^, and Asn^321^. Here, the residues Asp^362^ and Asn^321^ showed two double-hydrogen bond interactions in 58% and 55%, while for Asp^362^ and Asn^321^, hydrogen bonds were found in 35% and 64% of the total frames, respectively. The residue Val^320^ showed relatively high hydrogen bond interaction, with 61% observed out of the total frames, while Arg^3^, Lys^358^, and Leu^360^ showed relatively low hydrogen bond possibility, with 39%, 31%, and 30% of the total number of frames. The complex formed by Nigcollin C showed only two hydrogen bond interactions with the residues Lys^358^ and Val^320^, while it was observed that Lys^358^ had a direct hydrogen bond in 97% of the total frames and a water-mediated hydrogen bond with 34% of the total frames. Here, Val^320^ also showed water-mediated hydrogen bond interaction with 80% of the total frames. Interestingly, the protein–ligand complex with the compound NPA024545 showed only one direct hydrogen bond with residue Tyr^4^, shown in 31% of the frames, while Asp^362^, Asn^321^, and Arg^3^ showed water-mediated hydrogen bonds in 63%, 45%, and 33% of the total frames during the simulation. Additionally, the compound NPA024545 also showed π-π stacking with residue Phe^359^ in 31% of the total frames. During the simulation, Vaccinol M showed only two direct hydrogen bonds, with the residues Leu^360^ and Asp^362^ in 63% and 95% of the total frames, respectively. 

### 3.6. MM/GBSA Analysis

Furthermore, the trajectories during the last 10 ns of each complex for all five ligands, Gallicynoic Acid F, H2 Erythro-Neopterin, Nigcollin C, NPA024545, and Vaccinol M, were analyzed using MM/GBSA (Molecular Mechanics Generalized Born Surface Area) binding free energy calculation to estimate the binding affinities of the respective ligands at the active site. Total ΔG_Bind_ and individual energy components computed from the simulation trajectories were plotted in Supplementary Figure S8 and given in numerical values in Table 3. The graphs of the different energy components of the MM/GBSA were plotted to summarize the MM/GBSA scores. Here, Table 3 shows the energy components for the top five hits and the reference ligand. The bar plots for the energy values ΔG_Bind_, ΔG_Bind- coulomb_, ΔG_Bind-covalent_, ΔG_Bind-Hbond_, ΔG_Bind-Lipo_, ΔG_Bind-SolvGB,_ ΔG_Bind-vdW_, and ΔG_Bind-Ligstrain_ are shown in Supplementary Figure S8 for the top five hits. Similarly, the energy component bar plots for the native ligand are shown in Supplementary Figure S10. As shown in Supplementary Figure S8 and Table 3, the ΔGBind Coulomb and ΔGBind-vdw contributed the most to the binding of the respective complexes. In contrast, ΔGBind-Covalent and ΔGBind-_SolvGB_ showed positive binding energy for the docked complexes. Here, Gallicynoic Acid F and H2-Erythro-Neopterin docked complexes showed considerably lower binding free energy (ΔG_Bind_ < −61 kcal/mol) compared to other docked complexes, including the TTP-1671 reference complex (ΔG_Bind_ = −53.86 kcal/mol). In both the most energetically favored complexes, coulombic interaction contributed most. Nigcollin C, NPA024545, and Vaccinol M compound complexes showed relatively higher binding free energy with −49.31 kcal/mol, −41.27 kcal/mol, and −41.40 kcal/mol, respectively. Moreover, the compound Gallicynoic Acid F showed a ΔG_Bind-Hbond_ of −4.30 ± 0.69 kcal/mol, while the minimum was observed for the compound H2-Erythro-Neopterin, with a ΔG_Bind-Hbond_ of −4.76 kcal/mol. The rest of the top five compounds, Nigcollin C, NPA024545, and Vaccinol, showed relatively higher ΔG_Bind Hbond_, including the reference ligand, TTP-6171, in the range of −2.04 kcal/mol to −1 kcal/mol. 

### 3.7. Principal Component Analysis (PCA) 

**Figure 8:**
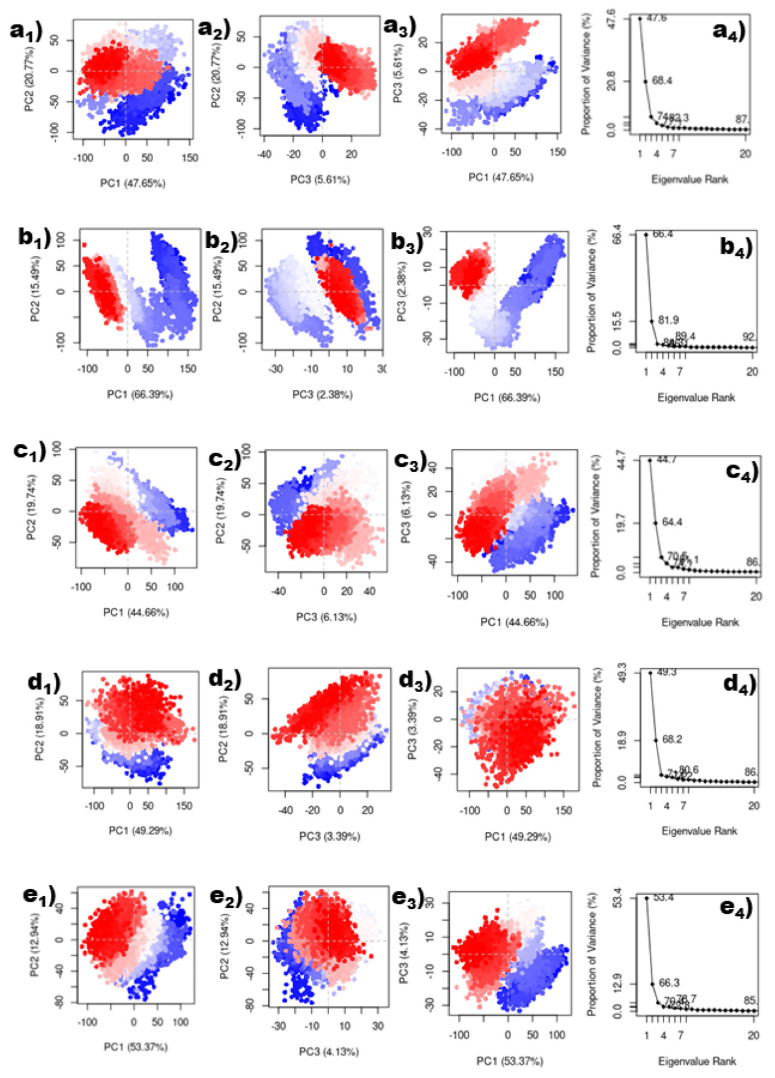
Principal component analysis of top five compounds. (**a1**–**a4**) Gallicynoic Acid F, (**b1**–**b4**) H2-Erythro-Neopterin, (**c1**–**c4**) Nigcollin C, (**d1**–**d4**) NPA024545, and (**e1**–**e4**) Vaccinol.

In this study, the protein’s conformational mobility upon binding to the top five hits was later determined using principal component analysis (PCA). PCA was also computed for the reference chemical for comparative analysis. A protein molecule exhibited high-dimensional mobility in the system during the simulation. PCA separates dimensions into their basic core constituents. The first three key elements (eigen vectors) for each protein–ligand complex were examined in this case. These were assumed to be the structural motions of the protein obtained from the simulation that are statistically significant. Similarly, for the reference complex, the top three primary components were plotted in Supplementary data Figure S9. The PCA analysis for the complexes of the top five hits and the reference are shown in Figure 8. Compounds Gallicynoic Acid F, H2-Erythro-Neopterin, Nigcollin C, NPA024545, and Vaccinol showed 74.03%, 84.26%, 70.53%, 71.59%, and 70.44% movement coverage collectively in their first three principal components. PCA plots shown in Figure 8 and Supplementary Figure S9 had multiple data points, where each point represented the conformation of the protein. A color gradient is shown in the plots, indicating the initial to final stages of the simulation (blue–red). The compound H2-Erythro-Neopterin showed high dispersion of PC1 compared to all other four compounds. Here, compound Nigcollin C showed a relatively lower conformational variation of all three principal components. The reference ligand showed 50.76% movement coverage in its first three principal components. The reference ligand had the most compact variation for PC3 and PC2, while PC1 had a higher relative variation, as shown by the dispersion in the graphs in Supplementary Figure S9.

## 4. Conclusions

Monkeypox is a newly developed infection that is diagnosed using serological and genomic analysis. It has the potential to spread from animal to human, and then from human to human, via zoonotic reservoirs. This disease’s symptoms resemble smallpox and chickenpox. The proteinase of monkeypox virus (MPXV) plays a critical role in the viral replication process. Thus, proteinase is considered as a major drug target to inhibit the growth of MPXV. In this proteinase of MPXV, the catalytic site residues Cys^328^ and His^241^ are responsible for the enzymatic activity. In this study, an in silico drug design approach was used to screen natural compounds against the proteinase of MPXV. The structure of the MPXV proteinase has not been solved experimentally. Hence, the 3D structure was modeled using Alphafold Colab v2.1.0. The catalytic residue Cys^328^ was found in the predicted binding pocket of the protein. Later, HTVS was performed with 32,552 compounds, followed by a docking process using the Glide Schrodinger suite. This resulted in 41 highly scored compounds that further allowed for the selection of the top five hits. Gallicynoic Acid F, H2-Erythro-Neopterin, Nigcollin C, NPA24545, and Vaccinol M were the compounds that showed the best docking energies. Gallicynoic Acid F and NPA024545 showed the highest number of hydrogen bonds with five residues of the protein. This formed their stable complexes. In these top five compounds, Vaccinol M exhibited the most consistent and stable RMSD in the range of 2 Å to 3 Å for the 100 ns MD simulation. However, the ligands Gallicynoic Acid F and H2-Erythro-Neopterin showed the lowest MM/GBSA binding free energies of −61.42 kcal/mol and −61.09 kcal/mol, respectively. The native ligand TTP-6171 was also compared with the top five hit compounds. The native ligand showed a ΔG_Bind_ of −53.86 kcal/mol, which was higher than the two compounds Gallicynoic Acid F and H2-Erythro-Neopterin. Overall, the study demonstrated the stronger binding of the top five hits compared to the native ligand. Moreover, Gallicynoic Acid F and H2-Erythro-Neopterin outperformed other hit compounds and showed the maximum potential to create inhibitory action against MPXV cysteine protease. 

## Figures and Tables

**Table 1:** List of top 41 NP_ATLAS database compounds screened against the binding pocket of the modeled 3D structure of MPXV proteinase.

Compound ID	Binding Scores (Glide XP)
NPA002071	−10.56
NPA000530	−9.64
NPA029767	−9.25
NPA024545	−9.19
NPA030378	−8.98
NPA006097	−8.96
NPA021601	−8.85
NPA016675	−8.76
NPA013579	−8.74
NPA011274	−8.62
NPA029767	−8.60
NPA018278	−8.56
NPA000223	−8.51
NPA013579	−8.49
NPA027193	−8.48
NPA018278	−8.48
NPA022559	−8.38
NPA016067	−8.35
NPA018278	−8.29
NPA001793	−8.26
NPA011289	−8.20
NPA018446	−8.12
NPA014034	−8.03
NPA032003	−8.01
NPA008964	−7.98
NPA014740	−7.93
NPA003368	−7.92
NPA018953	−7.91
NPA002699	−7.88
NPA027193	−7.84
NPA020009	−7.83
NPA022559	−7.81
NPA024544	−7.75
NPA020000	−7.68
NPA020183	−7.67
NPA003300	−7.66
NPA010406	−7.65
NPA005638	−7.62
NPA022152	−7.60
NPA002591	−7.59
NPA009242	−7.59

**Table 2:** List of interacting residues and type of interactions from cysteine protease protein of MPXV with the top five hits and * reference native ligand molecule.

S.No.	Complex	H-Bond	Hydrophobic	Polar	π-π Stacking/ π-π Cation	Positive	Negative
**1**	Proteinase–Gallicynoic Acid F	Arg^3^, Tyr^4^ Lys^358^ Leu^360^, Asp^362^	Met^1^, Tyr^4^, Leu^7^, Phe^17^ Leu^21^, Met^136^, Phe^278^, Val^320^, Leu^323^, Phe^357^, Phe^359^, Leu^360^,	Thr^18^, Ser^279^, Asn^321^, Gln^322^	Lys^358^	Arg^3^, Lys^358^	Glu^2^, Asp^6^, Asp^362^
**2**	Proteinase–H2-Erythro-Neopterin	Arg^3^, Tyr^4^, Gln^322^, Lys^358^, Leu^360^	Tyr^4^, Leu^7^, Phe^278^, Val^320^, Leu^323^, Phe^357^, Phe^359^, Leu^360^, Ala^361^	Thr^18^, Asn^321^, Gln^322^	Tyr^4^	Arg^3^, Lys^358^	Glu^319^, Asp^362^
**3**	Proteinase–Nigcollin C	Arg^3^, Val^320^	Met^1^, Tyr^4^,Leu^7^, Phe^17^ Leu^21^, Met^136^, Phe^278^, Val^320^, Leu^323^, Phe^357^, Phe^359^, Leu^360^, Ala^361^	Thr^18^, Asn^321^, Gln^322^	--	Arg^3^, Lys^358^	Glu^2^, Asp^6^, Glu^319^
**4**	Proteinase–NPA024545	Arg^3^, Tyr^4^ Asp^6^, Phe^357^, Lys^358^, Leu^360^, Asp^362^	Met^1^, Tyr^4^, Leu^7^, Phe^17^ Leu^21^, Phe^278^, Val^320^, Leu^323^, Phe^357^, Phe^359^, Leu^360^, Ala^361^	Thr^18^, Asn^321^, Gln^322^	--	Arg^3^, Lys^358^	Asp^6^, Asp^362^
**5**	Proteinase–Vaccinol M	Met^1^, Arg^3^ Lys^358^, Leu^360^	Met^1^,Tyr^4^,Leu^7^, Phe^278^, Val^320^, Leu^323^, Phe^357^, Phe^359^, Leu^360^, Ala^361^	Thr^18^, Asn^321^, Gln^322^	--	Arg^3^, Lys^358^	Glu^2^, Asp^362^
**6**	* Proteinase–TTP-6171	**--**	**Leu^27^, Ile^34^,** **Val^36^, Leu^40^** **Phe^356^, Phe^368^, Ile^371^, Tyr^393^**	**Ser^26^, Asn^33^, Ser^37^**	**Hip^23^/Hip^23^**	**Hip^23^, Lys^364^ Lys^394^**	**Asp^35^** **Glu^397^**

**Table 3:** Total Molecular Mechanics Generalized Born Surface Area (MM/GBSA) binding free energy (kcal/mol) values computed for the MPXV docked with top five screened compound complexes. * reference native ligand molecule.

MM/GBSA Components	Gallicynoic Acid F	H2-Erythro-Neopterin	Nigcollin C	NPA024545	Vaccinol M	* TTP-6171
**ΔGBind**	−61.42 ± 5.66	−61.09 ± 3.28	−49.31 ± 1.98	−41.27 ± 10.44	−41.40 ± 5.06	−53.86 ± 6.50
**ΔGBind Coulomb**	−21.41 ± 12.68	−43.17 ± 3.83	−16.23 ± 2.60	−21.20 ± 9.12	−18.43 ± 3.95	−12.14 ± 4
**ΔGBind Covalent**	4.25 ± 0.76	0.99 ± 1.57	1.09 ± 1.06	2.16 ± 1.33	3.37 ± 0.69	4.37 ± 1.37
**ΔGBind Hbond**	−4.30 ± 0.69	−4.76 ± 0.24	−1.14 ± 0.19	−1.46 ± 0.81	−2.04 ± 0.37	−1 ± 0.27
**ΔGBind Lipo**	−18.66 ± 0.77	−4.10 ± 0.45	−15.74 ± 0.69	−12.58 ± 2.64	−15.51 ± 1.74	−23.70 ± 1.18
**ΔGBind Solv GB**	20.93 ± 11.07	22.97 ± 2.61	21.50 ± 2.35	30.34 ± 3.08	25.20 ± 1.61	30.72 ± 4.19
**ΔGBind vdW**	−42.22 ± 1.84	−31.99 ± 2.50	−38.80 ± 2.17	−38.15 ± 3.32	−33.09 ± 2.04	−49.37 ± 1.84
**ΔGBind Lig Strain Energy**	7.64 ± 1.29	3.30 ± 1.88	1.75 ± 0.99	4.61 ± 1.87	3.66 ± 0.91	4.31 ± 2.63

## Data Availability

The datasets used and/or analyzed during the current study are available from the corresponding author on reasonable request.

## Supplementary Materials

The following supporting information can be downloaded at: https://www.mdpi.com/article/10.3390/v15010251/s1. Figure S1. 2D structure representation of the native ligand (TTP-6171); Figure S2. The viral protein structural interaction with its native ligand TTP-6171 in (a) 3D and (b) 2D format; Figure S3. (a) RMSD of the viral protein with respect to its native ligand (b) RMSF of the viral protein from the protein-native ligand complex (c) RMSF of the ligand from the protein-native ligand complex; Figure S4. RMSF of the viral protein from the protein-native ligand complex (a) Gallicynoic Acid F (b) H2 Erythro-Neopterin (c) Nigcollin C (d) NPA024545 (e) Vaccinol M extracted from the total 100 ns MD simulation; Figure S5. RMSF of the ligand from the protein-native ligand complex (a)Gallicynoic Acid F (b) H2 Erythro-Neopterin (c) Nigcollin C (d) NPA024545 (e) Vaccinol M extracted from the total 100 ns MD simulation; Figure S6. (a) Protein-ligand contact mapping of the protein-native ligand complex, (b) Ligand-Protein interaction profiling extracted from 100 ns simulation time; Figure S7. First and last pose of the protein-native ligand complex extracted from 100ns simulation; Figure S8. Graphical representation of free binding energy of the selected natural compounds, (a) Gallicynoic Acid F, (b) H2-Erythro-Neopterin, (c) Nigcollin C, (d) NPA024545, (e) Vaccinol M; Figure S9. Principal component analysis of the protein-native ligand complex extracted from 100ns simulation; Figure S10. Free binding energy calculation of protein-native ligand complex.
