# Supplementary material for: Exploration of Microbially Derived Natural Compounds against Monkeypox Virus as Viral Core Cysteine Proteinase Inhibitors
Source: Viruses. 2023 Jan 16;15(1):251. doi: 10.3390/v15010251 (PMC9861291; doi:10.3390/v15010251)
Supplement: Supplementary file 1 [file viruses-15-00251-s001.zip › viruses-2135603-supplementary.pdf]

Identification of potential inhibitor from NP-ATLAS Database against Monkeypox Proteinase

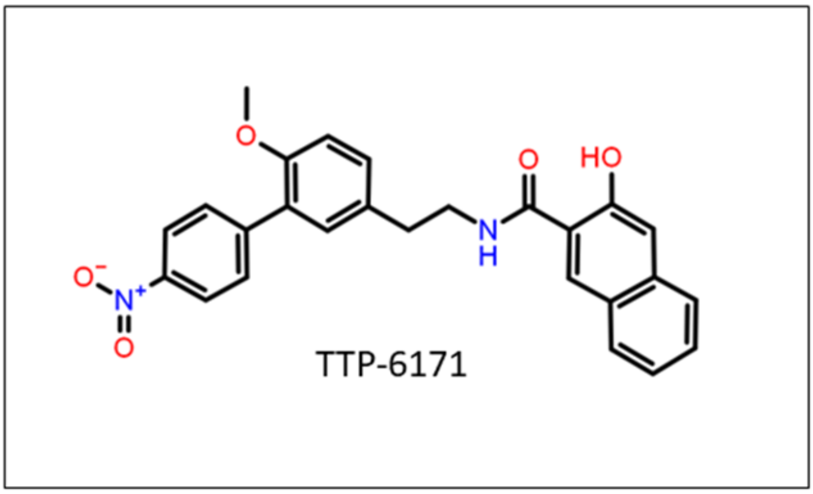

Figure S1. 2D structure representation of the native ligand (TTP-6171).

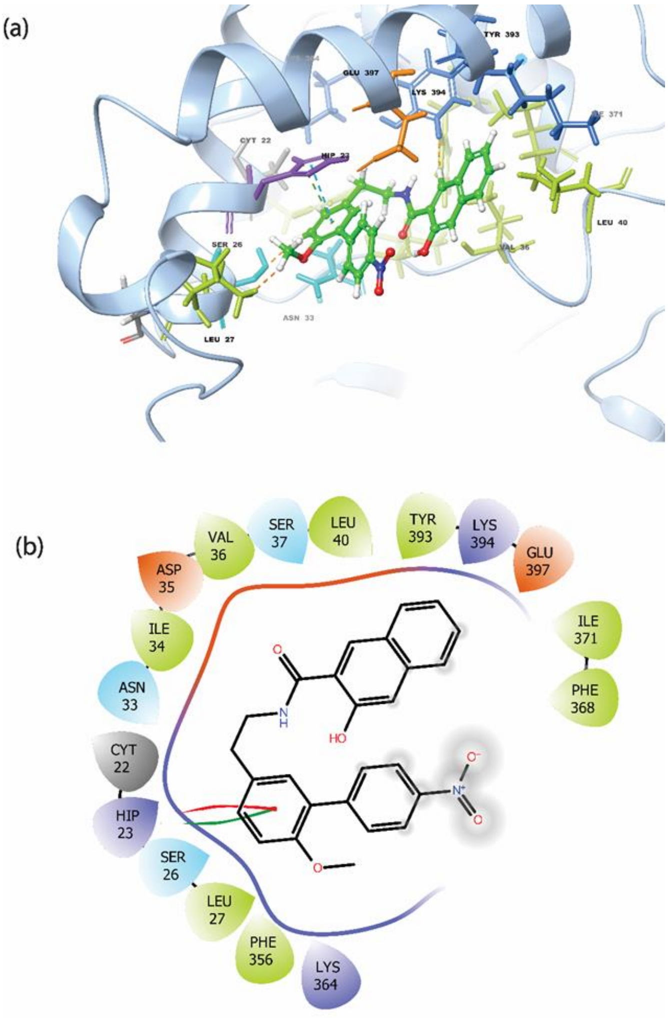

Figure S2. The viral protein structural interaction with its native ligand TTP-6171 in (a) 3D and (b) 2D format.

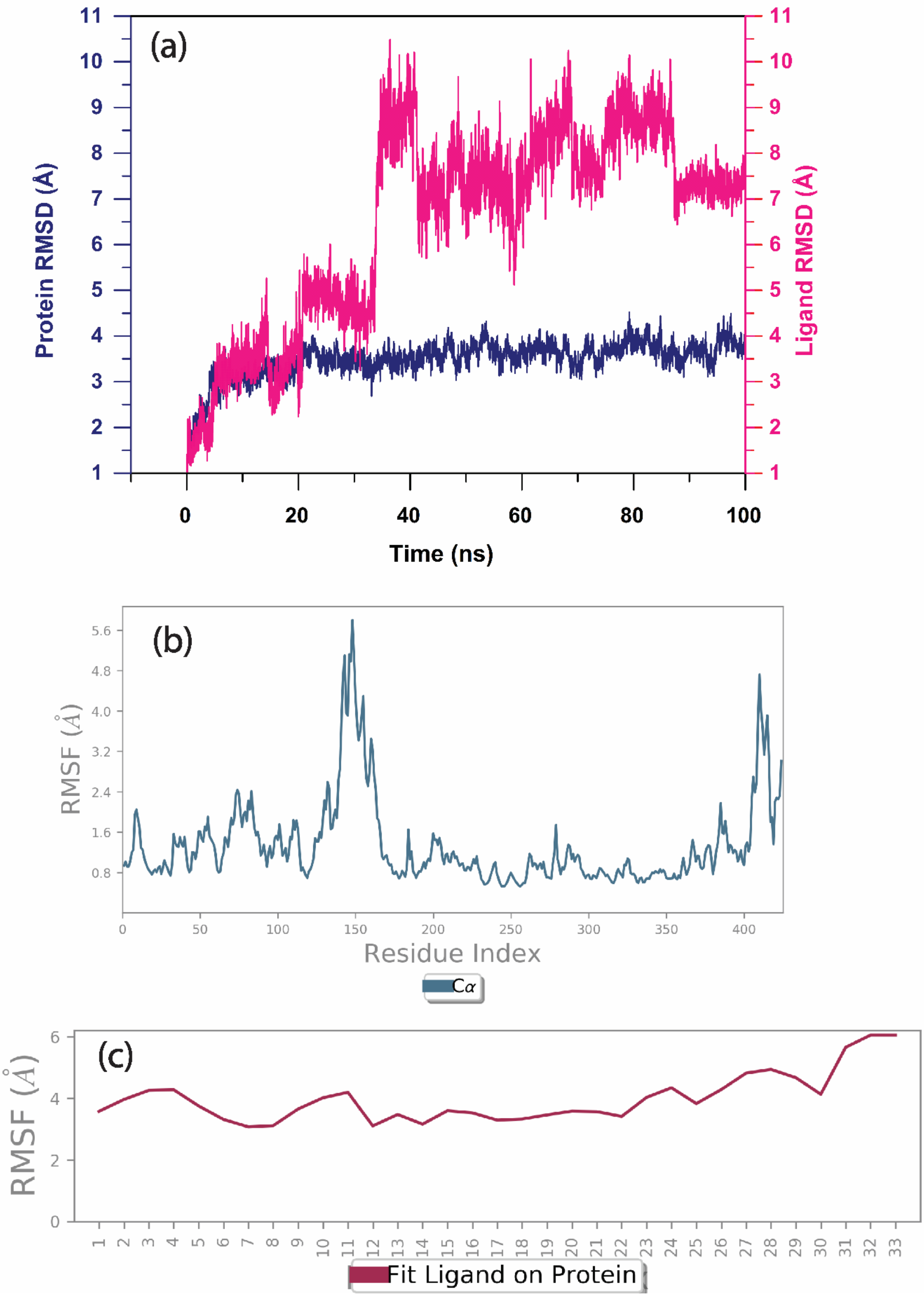

Figure S3. (a) RMSD of the viral protein with respect to its native ligand (b) RMSF of the viral protein from the protein-native ligand complex (c) RMSF of the ligand from the protein-native ligand complex.

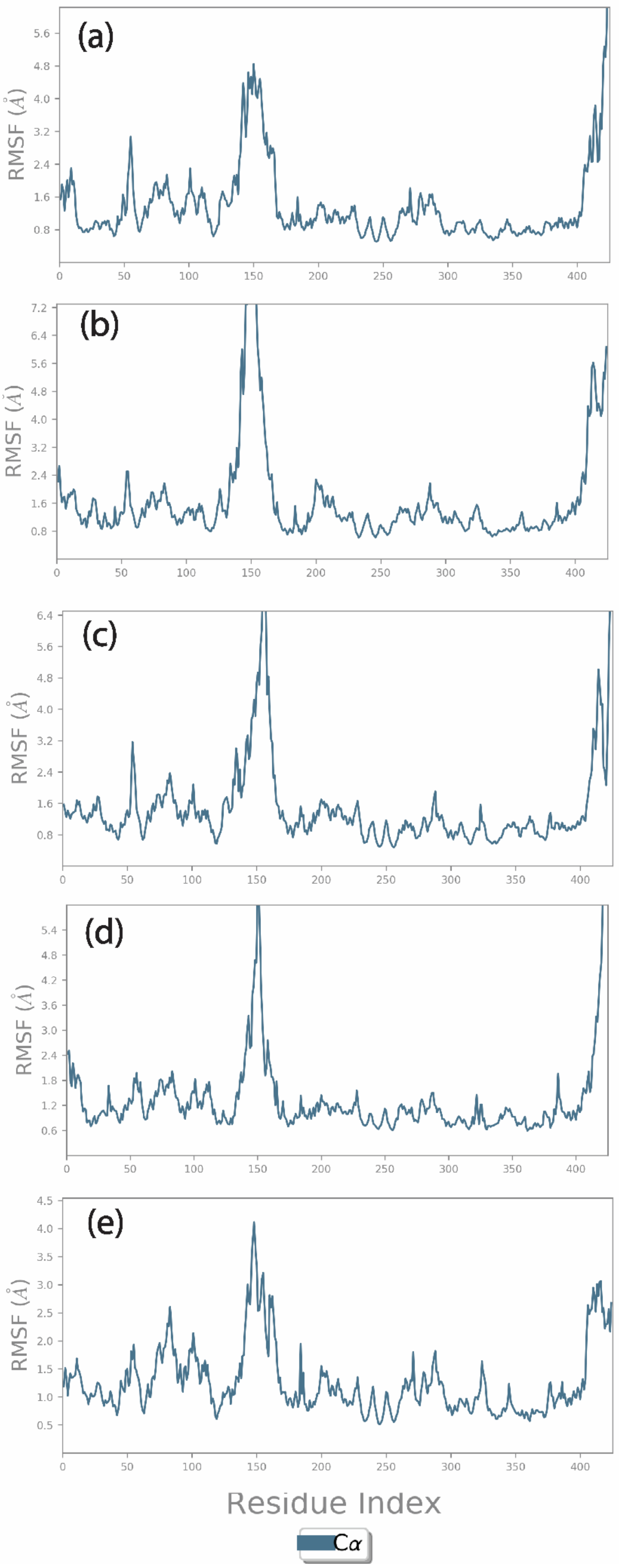

Figure S4. RMSF of the viral protein from the protein-native ligand complex (a) Gallicynoic Acid F (b) H2 Erythro-Neopterin (c) Nigcollin C (d) NPA024545 (e) Vaccinol M extracted from the total 100 ns MD simulation

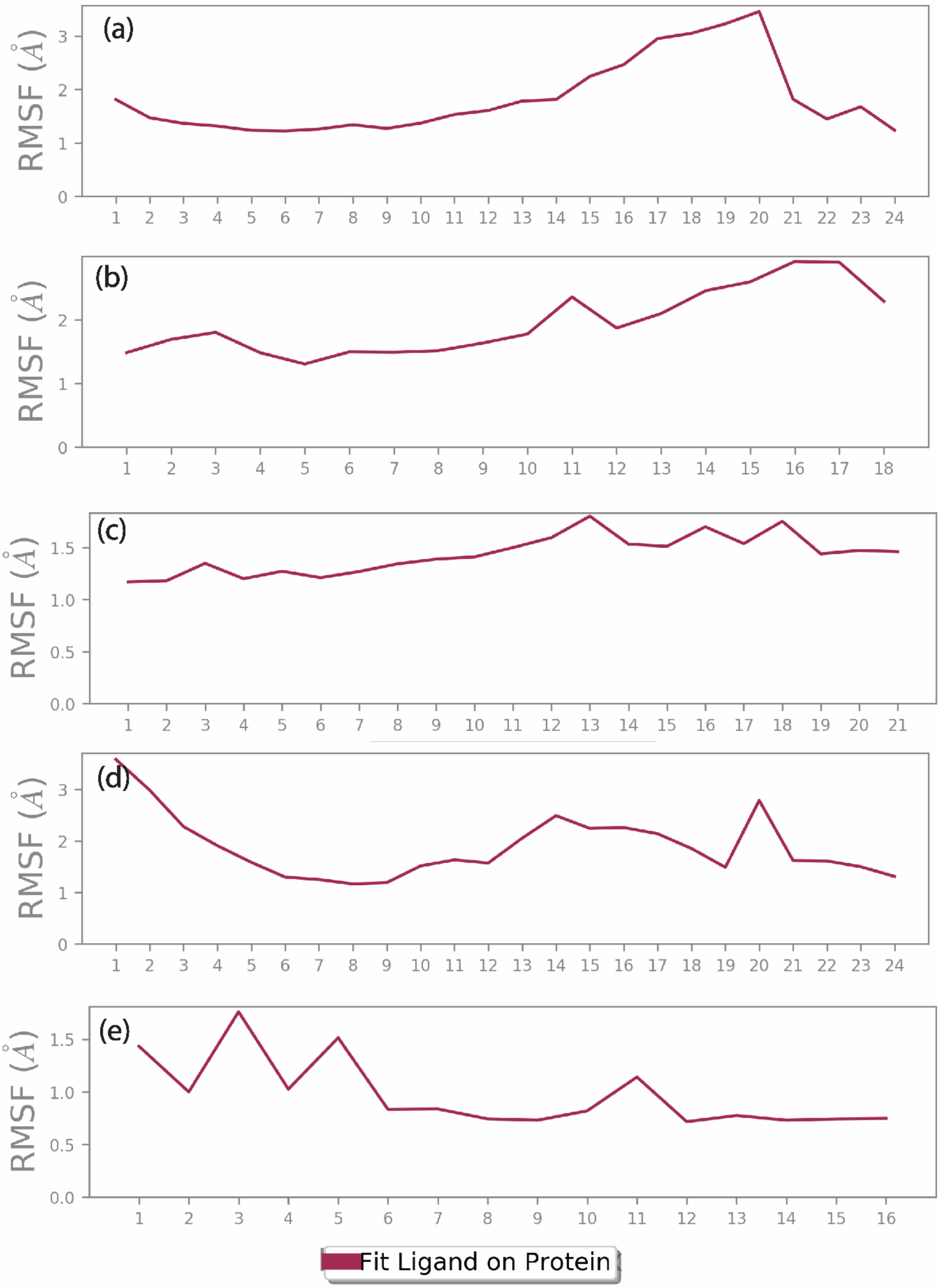

Figure S5. RMSF of the ligand from the protein-native ligand complex (a)Gallicynoic Acid F (b) H2 Erythro-Neopterin (c) Nigcollin C (d) NPA024545 (e) Vaccinol M extracted from the total 100 ns MD simulation

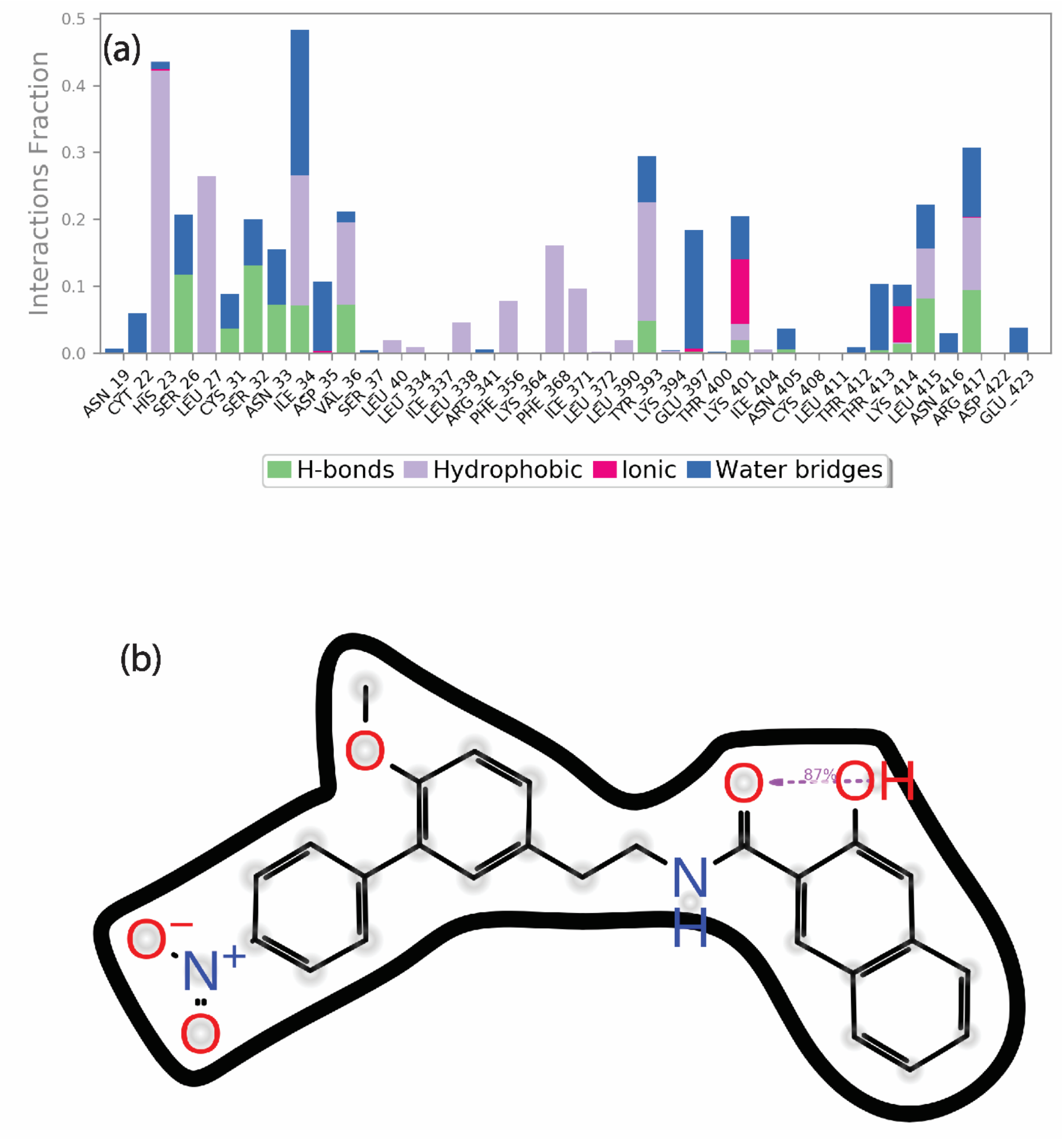

Figure S6. (a) Protein-ligand contact mapping of the protein-native ligand complex, (b) Ligand-Protein interaction profiling extracted from 100 ns simulation time

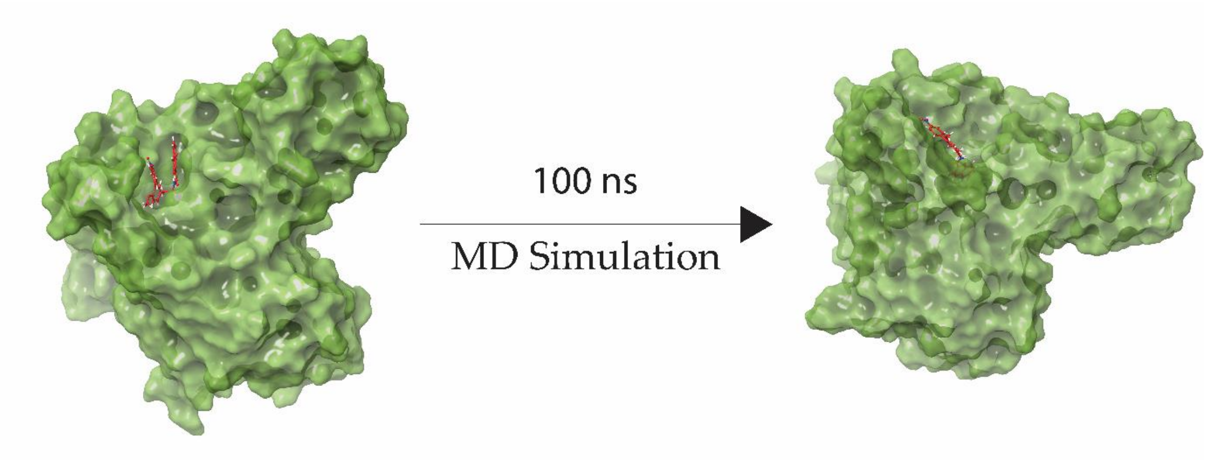

Figure S7. First and last pose of the protein-native ligand complex extracted from 100ns simulation.

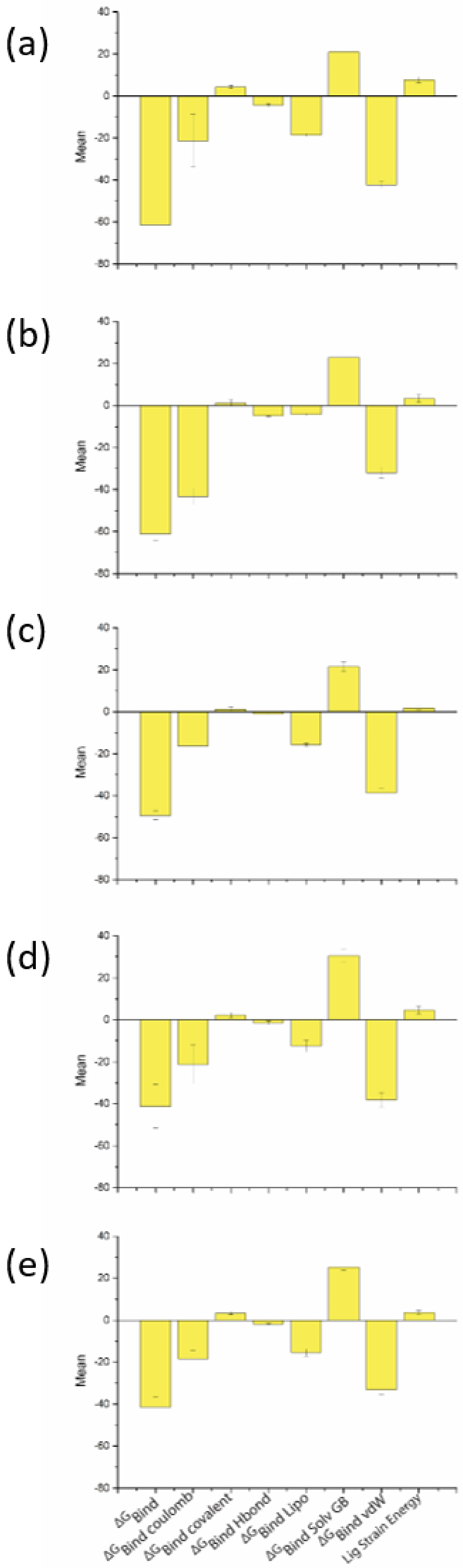

Figure S8. Graphical representation of free binding energy of the selected natural compounds, (a) Gallicynoic Acid F, (b) H2-Erythro-Neopterin, (c) Nigcollin C, (d) NPA024545, (e) Vaccinol M

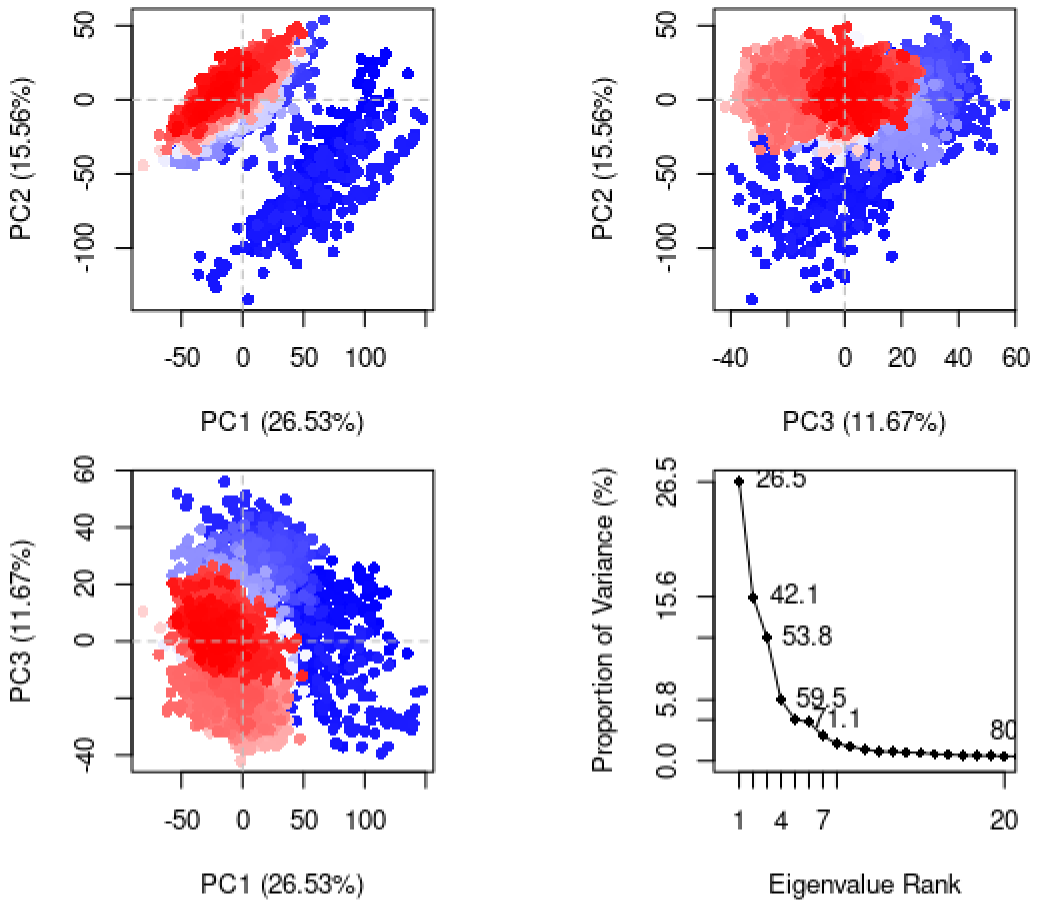

Figure S9. Principal component analysis of the protein-native ligand complex extracted from 100ns simulation.

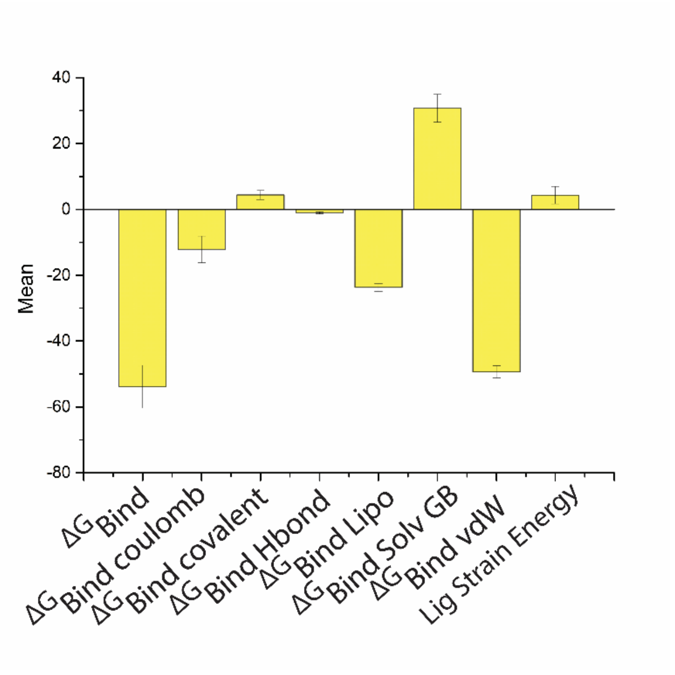

Figure S10. Free binding energy calculation of protein-native ligand complex
